# MicroRNA-96-3p promotes metastasis of papillary thyroid cancer through targeting SDHB

**DOI:** 10.1186/s12935-019-1003-y

**Published:** 2019-11-12

**Authors:** Xupeng Zhao, Yingjie Li, Yong Zhou

**Affiliations:** 1 Department of Fourth General Surgery, The Fourth Affiliated Hospital of China Medical University, Shenyang, Liaoning 110032 China; 2 Department of Sixth General Surgery, The Fourth Affiliated Hospital of China Medical University, Shenyang, Liaoning 110032 China

**Keywords:** MicroRNA-96-3p, Papillary thyroid cancer, SDHB, AKT pathway

## Abstract

**Background:**

MicroRNA (MiRNA) is a small non-coding RNA which is implicated in a cohort of biological function in cancer, including proliferation, metastasis, apoptosis and invasion. MiR-96 has been reported to be involved in many cancers, including papillary thyroid cancer. However, the role of miR-96-3p in papillary thyroid cancer metastasis is still unclear.

**Methods:**

qRT-PCR is used to detect the level of miR-96-3p and mRNA of SDHB in PTC tissues and cell lines. Western blot assays are used to verify the protein expression of SDHB. The transwell assays are performed to identify the migration ability of PTC cell lines. Moreover, dual-luciferase 3′-UTR reporter assays are chosen to illuminate the direct target of miR-96-3p.

**Results:**

The relative miR-96-3p upregulate in PTC tissues and three PTC cell lines (B-CPAP, K-1 and TPC-1 cells) while the relative SDHB is opposite. Our results revealed that the miR-96-3p promotes metastasis and invasion in PTC cell lines (K-1 and TPC-1 cells) by direct targeting SDHB and influence the downstream protein AKT.

**Conclusions:**

Taken together, the miR-96-3p is involved in PTC metastasis and invasion by direct targeting SDHB and the downstream molecule AKT and mTOR.

## Background

Papillary thyroid cancer (PTC), which belongs to a kind of differentiated thyroid cancer, is derived from the thyroid follicular epithelium. PTC has been reported to be accounted for the most cases among the thyroid cancers, which is regularly found in women and children [1, 2]. The increasing incidence of papillary thyroid cancer has been nearly universal in recently years [3–5]. Meanwhile, papillary thyroid cancer is the malignancy with fastest rising incidence in some regions. Among papillary thyroid cancers who are undertaken the surgery are generally associated with a favorable prognosis, however, the rate of recurrence can be still in 10–15% [4, 5]. Though distant metastases are involved in less than 10% of patients at diagnosis, the main cause of mortality is still arisen from the metastases [4, 6, 7]. Aberrant signaling pathways have been implicated in the onset, progression, and invasiveness of differentiated PTC and some evaluation methods have been established to avoid the tumor metastases according for these founded signaling pathways, but, the recurrence of papillary thyroid cancer is still in high rate [3, 8]. Thus, it is critical to develop accurate risk evaluation methods with the aim of preventing the recurrence of thyroid cancer.

Succinate dehydrogenase (SDH), a heterotetrametric complex, which includes SDHA, SDHB, SDHC and SDHD is an important citric acid cycle enzyme in mitochondria [9–11]. SDH is engaged in the oxidation of succinate to fumarate and in the electron transport [12, 13]. As the catalytic core component of SDH, SDHB dysfunction has been proved to be implicated in the attenuating oxidative phosphorylation and involved in couple of cancers [9, 14, 15]. Accumulating researches pointed that the loss of SDHB function was associated with invasion and metastasis [16, 17]. For example, SDHB deficiency promotes TGFβ-mediated invasion and metastasis of colorectal cancer through transcriptional repression SNAIL1–SMAD3/4 complex [17]. SDHB-mutated PPGL display a hypermethylator phenotype associated with hallmarks of epithelial-to-mesenchymal transition (EMT). SDHB deficient cells exhibit a metastatic phenotype as highlighted by increased individual cell migration (characterized by faster motility and increased persistence) as well as high invasive and adhesion abilities [18]. However, the mechanism of SDHB associated with the papillary thyroid cancer is poorly understood.

MicroRNAs (MiRNAs) are small, non-protein-coding RNAs which can regulate the gene expression post-transcriptionally by binding to mRNA 3′ untranslated region (3′UTR), leading to translational repression or mRNA degradation [19–21]. It has been revealed that most of human genes are regulated by miRNAs [22, 23]. More importantly, over 50% of the genes regulated by miRNAs are involved in cancer proliferation, metastasis, differentiation and apoptosis [22, 24]. Recently, vast arrays of attention have been thrown to miRNAs for their playing a vital role in malignant transformation and tumorigenesis in cancer patients [25–27]. Couples of researches had reported that miR-96 was dramatically up-regulated in different kind of tumors, including breast cancer, prostate cancer, bladder cancer and hepatocellular carcinoma [28–30]. For example, miR96 promote tumor invasion in colorectal cancer via RECK [31]. Moreover, overexpression of miR-96-5p inhibits autophagy and apoptosis and enhances the proliferation, migration and invasiveness of human cancer cells [32].Nevertheless, whether the miR-96-3p is involved in invasion and metastasis in papillary thyroid cancer remained poorly understood.

## Materials and methods

### Clinical specimens

Twenty-eight pairs of papillary thyroid carcinoma tissues and the adjacent normal tissue specimens were collected from the PTC patients who were undergone with surgical resections in the Department of Fourth general surgery, the Fourth Affiliated Hospital of China Medical University from January 2013 to October 2017. The clinical samples were immediately frozen in the liquid nitrogen after obtained from the patients and then stored at − 80 °C. In this research, Informed written consents were obtained from all the patients and the procedures were proved by the ethics committee of the Fourth Affiliated Hospital of China Medical University and the approval number is CMU-FAH2018032.

### Cell culture

The Human PTC cell line (B-CPAP) and the human thyroid epithelial cell line Nthy-ori 3-1 were bought from the Chinese Academy of Sciences (Shanghai, China) and the other two human PTC cell lines (K-1 and TPC-1 cells) and HEK293T cell line obtained from the European Collection of Cell Cultures (ECACC). Cultivation condition of K-1, TPC-1, B-CPAP and HEK293T lines contained with Dulbecco’s modified Eagle’s medium (DMEM; Invitrogen) and 10% fetal bovine serum (FBS; Gibco) and 1% penicillin/streptomycin at 37 °C with 5% CO2. The Nthy-ori 3-1 cells were cultured in RPMI-1640 medium (Invitrogen).

### Luciferase reporter assay

The HEK293T cell lines were seeded into 96-well plate per well of which contained 10,000 cells and 3′UTR-SDHB firefly luciferase reporter was co-transfected with miR-96-3P mimics (Gene-Pharma) by using Lipofectamine 2000. The luciferase activity was detected via dual-luciferase reporter assay system (Promega) according to the manufacturer’s instruction after 24 h. Wild-type and mutant targeted sequences of miR-96-3p in the protein SDHB 3′-UTR are as follow.

Mut: 5′…CUGUUUCCAUGCUAAUGUACUUU…

WT: 5′…CUGUUUCCAUGCUAAACAUGAUU…

miR-96-3p: 3′…GUAUAACCGUGACGUGUACUAA…

### Western blot analysis

The extracted protein after boiled for 6 min was separated by 10% SDS-PAGE (BioRad) and then transferred onto a PVDF membrane (Millipore, USA). Then the PVDF membrane is blocked with 5% fat-free milk for 60 min. Primary antibodies were incubated at 4 °C overnight. In the following day, the TBST is used to wash out the primary antibodies and the membranes are incubated with secondary antibodies. The primary antibodies used in the experiment were as follows: rabbit anti-human AKT (1:1000, Cell Signaling Technology #4691S), rabbit anti-human phospho-AKT (Ser473) (1:1000, Cell Signaling Technology #4060S), mouse anti-β-actin (1:2000, Santa Cruz #sc-47778) and mouse anti-SDHB antibody (1:1500, Abcam #ab14714).

### Transwell assay

The transwell assays are used to detect the migration and invasion of the PTC cell lines. Briefly, 24-well Boyden chamber with 60,000 TPC cells in the upper chamber is filled with serum-free DMEM with fibronectin (Roche) and Matrigel (BD Biosciences) for the invasion assays, while fibronectin only for migration. In lower chamber, was filled with 600 μL DMEM with 10% fetal bovine serum.

### Quantitative real-time PCR

Total RNA extractions from cell lines and frozen tissue specimens were conducted with TRIzol^®^ reagent (Invitrogen). The Takara Reverse Transcription System Kit (Takara Biotechnology Co. Ltd, Japan) were used to synthesize cDNA. The quantitative real-time reverse transcription polymerase chain reaction (qRT-PCR) was performed using the SYBR green premix kit (BioRad, Hercules, CA, USA). GAPDH and U6 was used as internal controls for SDHB and miR-96-3p respectively. The sequences of the primers were as follows:

MiR-96-3p: 5′-GCCCGCTTTGGCACTAGCACATT-3′ (Forward); 5′-GTGCAGGGTCCGAGGT-3′ (Reverse). SDHB: 5′-GACACCAACCTCAATAAGGTCTC-3′ (Forward); 5′-GGCTCAATGGATTTGTACTGTGC-3′ (Reverse). GAPDH: 5′-GGCACAGT-CAAGGCTGAGAATG-3′ (Forward), 5′-ATGGTGGTGAAGACGCCAGTA-3′ (Forward).

### Statistical analysis

All experiments were performed independently at least three times. Data are presented as the mean ± standard deviation (SD) and were analyzed using GraphPad Prism™, version 6.00 software (GraphPad, La Jolla, CA, USA). The Student’s t-test or oneway ANOVA was used to determine statistical significance of differences between two groups or among variant groups, respectively. A *p* value < 0.05 was considered statistically significant.

## Results

### MiR-96-3p in human PTC tissues compared with paired adjacent normal tissues and three PTC cell lines is significantly up-regulated

**Fig. 1:**
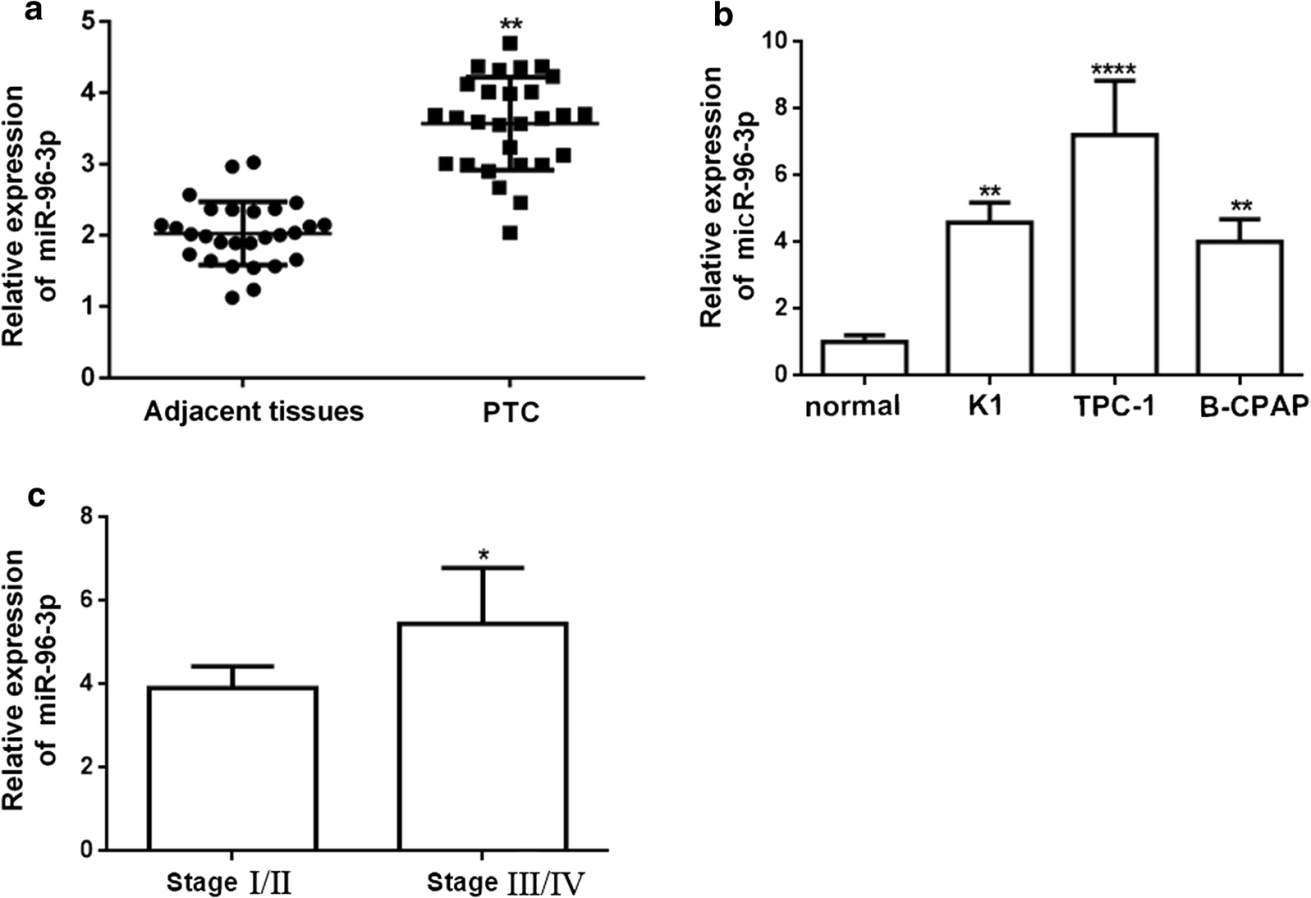
MiR-96-3p is up-regulated in the PTC tissues and the PTC cell lines. The relative miR-96-3p is dramatically increased in PTC tissues compared with adjacent normal thyroid tissues by real-time quantitative reverse transcription polymerase chain reaction (qRT-PCR) assay (n = 28; **a**). The relative miR-96-3p is significant up-regulated in PTC cell lines compared with a normal human thyroid epithelial cell line, Nthy-ori 3-1 (**b**). The relative expression of miR-96-3p in TNM stages III/IV is higher than that of stages I/II (n = 28; **c**). *p < 0.05, **p < 0.01, ***p < 0.001

To explore the function of miR-96-182-183 cluster in PTC, we collected clinical data and the corresponding PTC tissues and paired adjacent normal tissues of the 28 PTC patients. As shown in the Fig. 1a and Additional file 1: Figure S1, we detected that the relative expression of miR-96-3p in PTC patients was dramatically upregulated in the PTC tissues compared with adjacent paired normal tissues, while there were no obvious significant difference in the expression of miR-182, miR-183 and miR-96-5p in two groups. We then determined whether the miR-96-3p was also overexpression in the PTC cell lines (B-CPAP, K-1 and TPC-1 cells). In consistent with the results in vivo, the relative miR-96-3p was obviously up-regulated in all the three PTC cell lines (Fig. 1b). At the same time, we performed a clinical analysis with the all 28 PTC patients, the summarization of which was presented in Tables 1 and 2.

**Table 1:** Association between SDHB expression (PTC tissues over normal adjacent thyroid tissues) and clinical characteristics in papillary thyroid cancer (PTC)

Clinical characteristics	n	SDHB	*p*-value
Age (year)			
≤ 44	19	0.82	0.00089***
>44	9	1.52	
Sex			
Male	10	1.11	0.074
Female	18	1.32	
Multicentricity			
No	18	1.25	0.062
Yes	10	1.19	
Cervical LN metastasis			
No	11	1.42	0.009**
Yes	17	0.78	
TNM			
I/II	20	1.37	0.041*
III/IV	8	0.84	
Tumor size			
≤ 2 cm	23	1.21	0.056
> 2 cm	5	1.28	

* *p *< 0.05, ** *p *< 0.01, *** *p *< 0.001 compared with normal adjacent thyroid tissues

**Table 2:** Association between miR-96-3p relative expression (PTC tissues over normal adjacent thyroid tissues) and clinicopathological characteristics in papillary thyroid cancer (PTC)

Clinical characteristics	n	miR-96-3p	*p*-value
Age (year)			
≤ 44	19	5.365	0.053
> 44	9	4.123	
Sex			
Male	10	4.235	0.092
Female	18	4.112	
Multicentricity			
No	18	3.925	0.067
Yes	10	4.256	
Cervical LN metastasis			
No	11	4.968	0.004**
Yes	17	3.456	
TNM			
I/II	20	3.865	0.003**
III/IV	8	5.768	
Tumor size			
≤ 2 cm	23	4.098	0.071
> 2 cm	5	3.889	

* *p *< 0.05, **p < 0.01, ***p < 0.001 compared with normal adjacent thyroid tissues

Surprisingly, we found that the miR-96-3p was remarkably elevated in stage III/IV compared with stage I/II in the PTC patients (Fig. 1c). Thus, according to the above evidence, miR-96-3p may be embodied with advanced TNM stages and play a vital role in the PTC distant metastasis.

### MiR-96-3p increases the invasion and migration of PTC cell lines

**Fig. 2:**
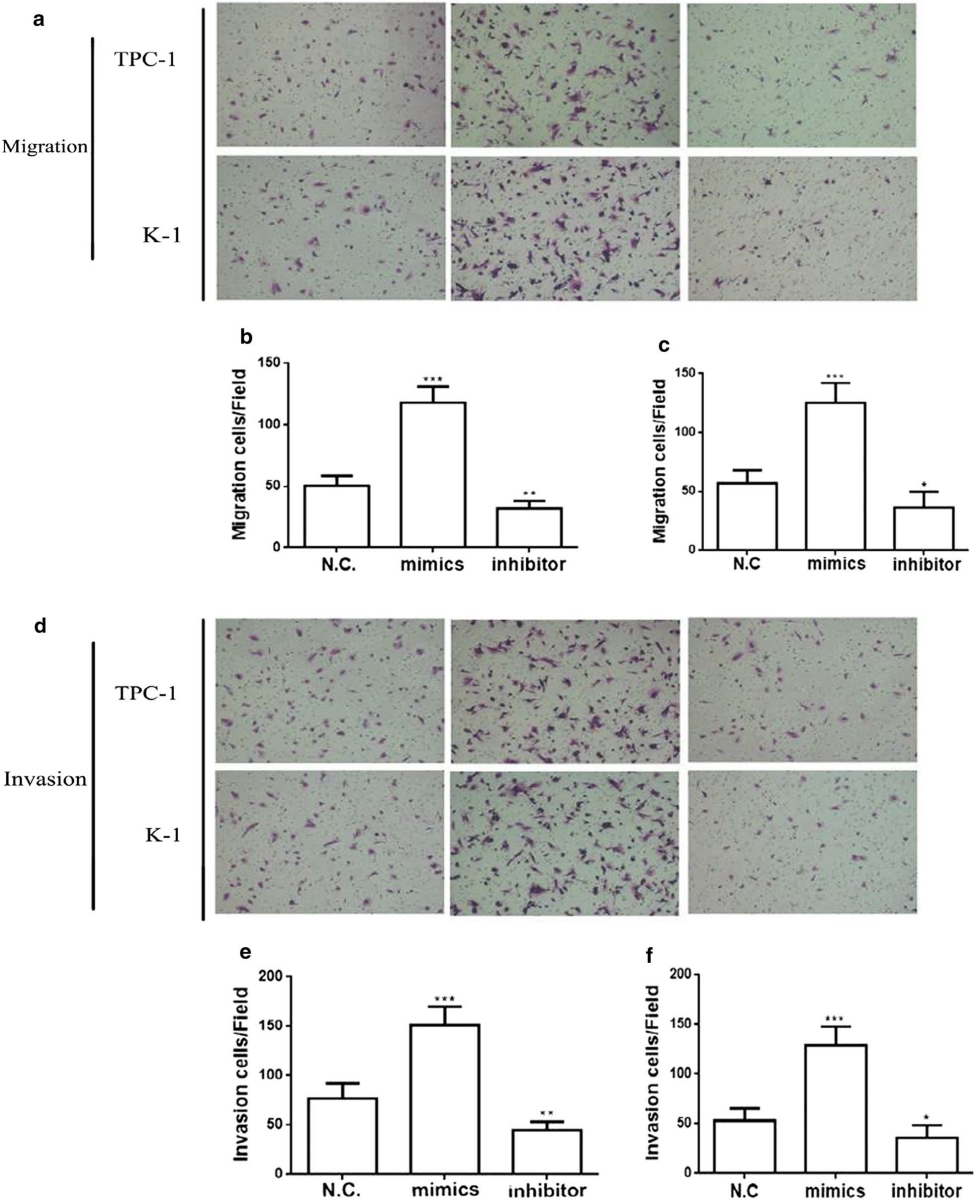
MiR-96-3p promotes human papillary thyroid cancer (PTC) cell migration and invasion. The effects of miR-96-3p in TPC-1 and K-1 are showed through performing transwell migration assays (**a**, **d**). The effects of miR-96-3p in TPC-1 and K-1 are showed through performing transwell migration assays (**b**, **c**). The effects of miR-96-3p in TPC-1 and K-1 are showed through performing transwell invasion assays (**e**, **f**) *p < 0.05, **p < 0.01, ***p < 0.001

To further identify whether miR-96-3p was involved in PTC distant metastasis, miR-96-3p mimics and inhibitor were transfected into PTC cell lines (K-1 and TPC-1 cells) for K-1 cell lines with relative higher of expression miR-96-3p and the B-CPAP and TPC-1 cell line with relative lower of expression miR-96-3p compared with the normal thyroid epithelial cell line, Nthy-ori3-1. At first, for TPC-1 cells and K-1 cells, we found that the addition of the mimics of miR-96-3p increased the cell proliferation and MMP-9 expression (Additional file 1: Figure S2). Matrigel-uncoated Transwell assays or Matrigel-coated Transwell assays were performed to determine the effect of miR-96-3p on the migration and invasion in the PTC cell lines. As shown in the Fig. 2a–c, Transwell assays without Matrigel indicated that overexpression of the miR-96-3p can promote the migration of TPC-1 and K-1 cells, while inhibiting the expression of the miR-96-3p can suppress the migration of K-1 and TPC-1 cells. Likewise, Transwell assays with Matrigel indicated that miR-96-3p mimics enhanced the invasion ability and the miR-96-3p inhibitor presented the reverse effects in the K-1 and TPC-1 cells (Fig. 2d–f).

Above all, the miR-96-3p can dramatically enhance the migration and invasion capability in K-1 and TPC-1 cells in vitro, these evidences may be in consistent with the observation in vivo that miR-96-3p was implicated in PTC distant metastasis.

### SDHB is down-regulated in human PTC tissues and PTC cell lines and miR-96-3p directly targets the 3′-untranslated regions (3′-UTRs) of SDHB

Having proved miR-96-3p involved in the migration and invasion in PTC, and then we urged to investigate the molecular mechanism. We use target scan to find the potential targets. Finally, we found the SDHB may be a putative target of the miR-96-3p. Additionally, there was also research reporting that the SDHB was involved in the PTC.

**Fig. 3:**
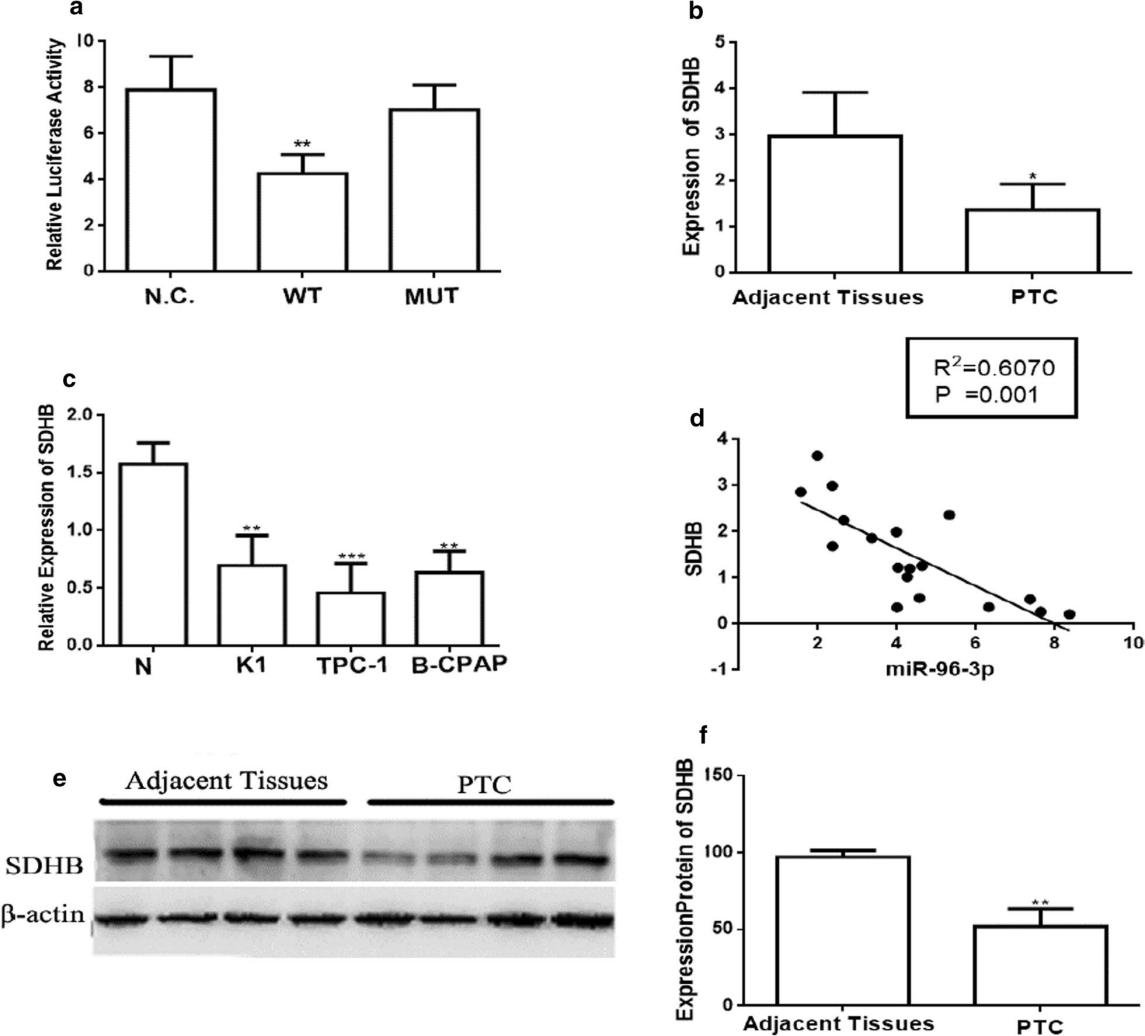
SDHB is a direct target of miR-96-3p. Luciferase report assays showed miR-96-3p targeted in the protein SDHB 3′-UTR in Wild-type and mutant (**a**). The relative expression mRNA of SDHB in adjacent tissues and PTC tissues in PTC patients (**b**). The relative expression mRNA of SDHB in the three PTC cell lines (**c**). The scatter plot showed an inverse correlation between SDHB mRNA and miR-96-3p expression in 2 (**d**). Protein levels of SDHB were overexpressed in PTC samples compared with adjacent normal thyroid tissues in PTC patients using western blot assay (**e**, **f**) *p < 0.05, **p < 0.01, ***p < 0.001

To investigate whether the SDHB was the direct target of miR-96-3p, luciferase reporter assay vectors with 3′-UTR of SDHB were constructed. As shown in Fig. 3a, Luciferase activity of SDHB3′-UTR was markedly suppressed by the overexpression of miR-96-3p. However, the mutant vectors showed no effects in luciferase activity by the overexpression of miR-96-3p. Given all these evidences, the miR-96-3p directly targets the 3′-untranslated regions (3′-UTRs) of SDHB.

Interestingly, as shown in the Fig. 3b, the results demonstrated the relative expression of SDHB was marked reduced in the PTC tissues compared with the paired adjacent normal tissues by qRT-PCR assay. Moreover, mRNA level of the SDHB in the three PTC cell lines (compared to the normal thyroid epithelial cell line) was remarkably reduced (Fig. 3c). To verify if the protein level of the SDHB had the similar phenomena, the Western blot analysis was used to analyze that of PTC tissues and the paired normal adjacent tissues of 28 PTC patients. The protein level of SDHB in the PTC tissues was decreased compared to the normal adjacent tissues (Fig. 3e, f).

### MiR-96-3p down regulates expression of SDHB and activates AKT/mTOR pathway

Having knowledge of the relative mRNA of SDHB in PTC patients and PTC cell lines, we choose to determine the downstream of SDHB signal pathway. It has been well established the AKT plays an important role in a cohort of cancer to metastasis. So we put a hypothesis the miR-96-3p targets SDHB and influence downstream protein AKT to promote metastasis.

**Fig. 4:**
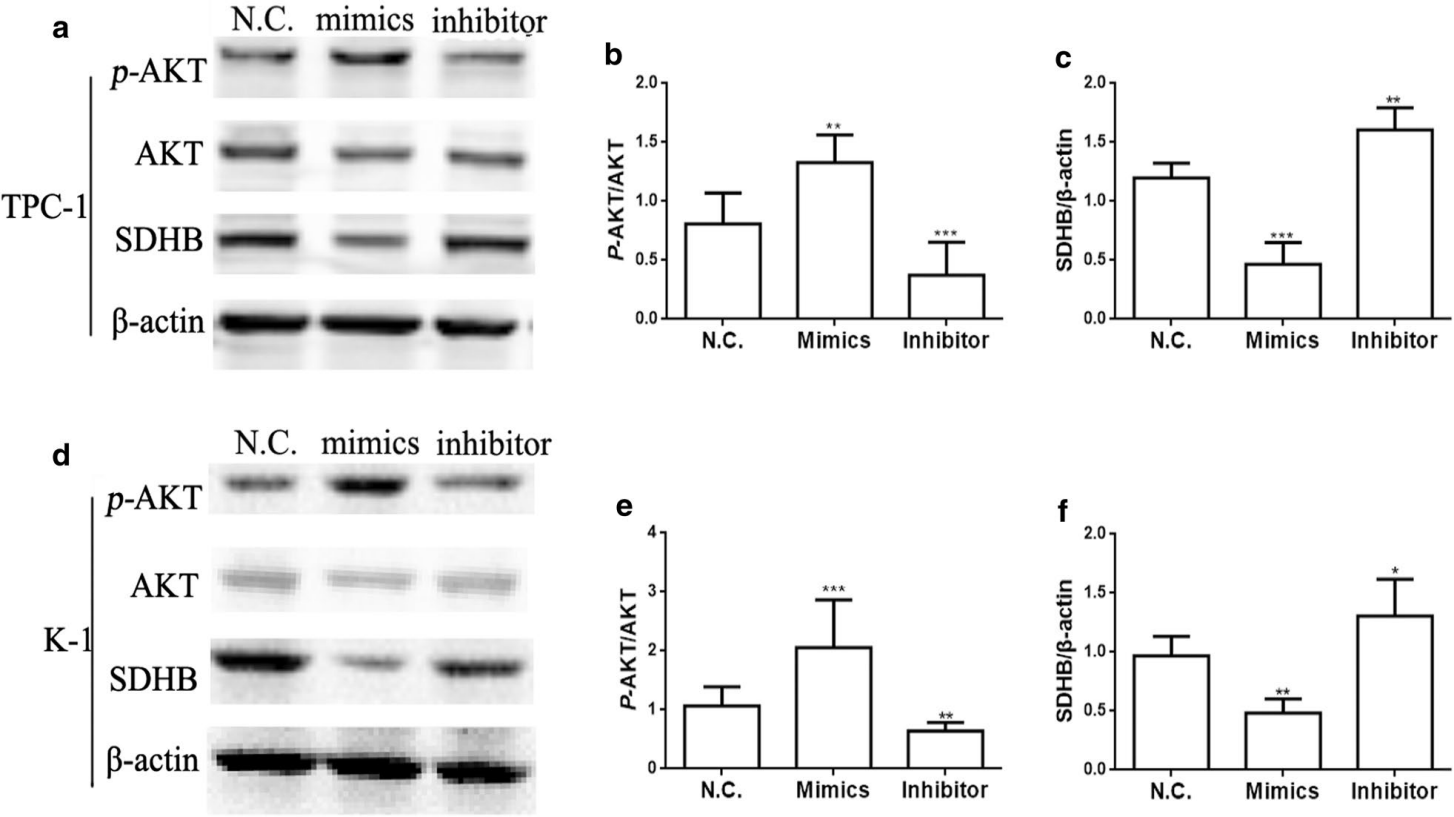
Activation of miR-96-3p up-regulated the *p*-AKT and down-regulated the SDHB. The expression of *p*-AKT in TPC-1 cell line of different group (**a**, **b**). The expression of SDHB in TPC-1 cell line of different group (**a**, **c**). The expression of *p*-AKT in K-1 cell line of different group (**d**, **e**). The expression of SDHB in K-1 cell line of different group (**d**, **f**). *p < 0.05, **p < 0.01, ***p < 0.001

Therefore, we use the western blot assay to evaluate the protein level of the SDHB and AKT pathway. The *p*-AKT and mTOR protein was remarkably decreased with miR-96-3p inhibitor while the *p*-AKT protein was substantially increased with miR-96-3p mimics in TPC-1 cells (Fig. 4a, b, Additional file 1: Figure S3). Conversely, the SDHB protein showed the reverse reaction with the miR-96-3p inhibitor and mimics (Fig. 4a, c). In addition, the effects of mimics and inhibitor of miR-96-3p on *p*-AKT, mTOR and SDHB in K-1 cell consistent with the TPC-1 cells (Fig. 4d–f, Additional file 1: Figure S3).

### Upregulation of the SDHB inhibits the miR-96-3p-mediated invasion and migration by decreasing the *p*-AKT in TPC-1 cells

**Fig. 5:**
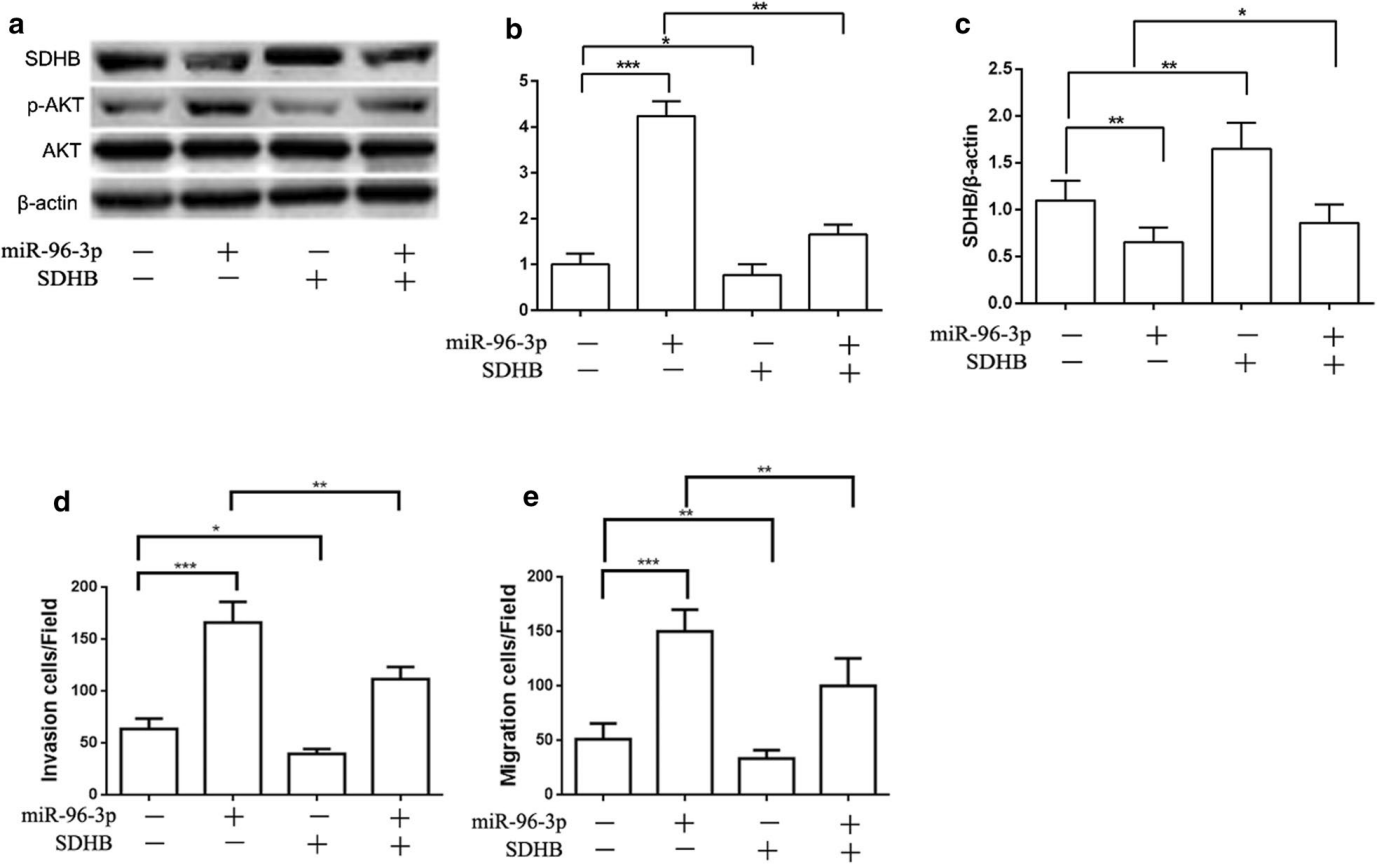
MiR-96-3p promote migration and invasion through SDHB/AKT pathway. The expression of SDHB, *p*-AKT in TPC-1 cells (**a**–**c**). The migration and invasion was tested in TPC-1 cells of different group (**d**, **e**). *p < 0.05, **p < 0.01, ***p < 0.001

Now that the miR-96-3p can downregulate the SDHB and promote the PTC cells invasion and migration in the PTC cell lines (K-1 and TPC-1 cells), we hypothesize the overexpression of the SDHB can reverse these effects of miR-96-3p in TPC-1 cells. So, we constructed SDHB overexpression vectors to verify the hypothesis. As shown in the Fig. 5a, the *p*-AKT expression is significantly increased with the miR-96-3p mimics but this phenomenon can be reversed after the SDHB overexpression. At the same time, the results of transwell assays showed overexpression of SDHB can reverse the miR-96-3p mimics promoting the invasion and migration in TPC-1 cells.

### Effects of miR-96-3p on the tumor volume in vivo

Compared with control group, we found the tumor volume decreased in the miR-96-3p group (Additional file 1: Figure S4). In PTC tissue, the SDHB expression was negatively correlated with the expression of miR-96-3p (Additional file 1: Figure S5).

## Discussion

Papillary thyroid cancer (PTC) is the most common thyroid cancers all over the world and its incidence and recurrence is still increasing these years [33, 34]. However, the mechanism of the PTC is remaining poor understood. Although miRNA is a kind of small non-coding RNA, the miRNA is involved in a vast array of the biological function, including proliferation, apoptosis, metastasis and invasion [35, 36]. MiR-96-3p has been reported to be a cancer-associated miRNA in many kinds of cancers [29, 37]. However, the function of the miR-96-3p involved in invasion and migration in PTC is still unknown.

Previous studies have indicated miR-96 may increase cancer cell proliferation and migration in bladder cancer and breast cancer [28, 30]. In this study, it was identified that the relative miR-96-3p was highly expressed in papillary thyroid cancer tissues compared with the paired benign tissues in PTC patients. In addition, patients who diagnosed with PTC in stage III/IV showed upregulation of miR-96-3p in PTC tissues compared with adjacent tissues. Simultaneously, the increased expression of miR-96-3p promoted the invasion and migration in three PTC cell lines. Furthermore, miR-96-3p inhibitors can abrogate the PTC cell invasiveness and migration.

Given to the miR-96-3p associated with the metastasis in vivo and the invasion and migration in vitro in papillary thyroid cancer. We endeavor to determine the mechanism of the effects of miR-96-3p on papillary thyroid cancer. There are researches indicated that SDHB involved in the metastasis of cancers. In this study, we revealed miR-96-3p directly targeted the SDHB and the upregulation of miR-96-3p and the downregulation of SDHB in the PTC tissues and PTC cell lines. Furthermore, the miR-96-3p mimics leaded to reducing the expression of SDHB in vitro while the miR-96-3p inhibitor leaded to reverse effects. Most importantly, over expression of SDHB can inhibit the migration and invasion induced by miR-96-3p, implicating the miR-96-3p/SDHB may play a key role in the process of the cancer and metastasis of the PTC.

To further verify the downstream molecular mechanism of the miR-96-3p/SDHB in metastasis in thyroid cancer, we focused on the AKT/mTOR pathway, which has been proved to be cellular biological function of the cancer [38–40]. Our results showed that the miR-96-3p mimics in PTC cell lines could downregulate the SDHB and upregulate the *p*-AKT and mTOR. The miR-96-3p inhibitor showed the opposite effects that indicated the miR-96-3p may promote the invasion and metastasis in the PTC.

**Fig. 6:**
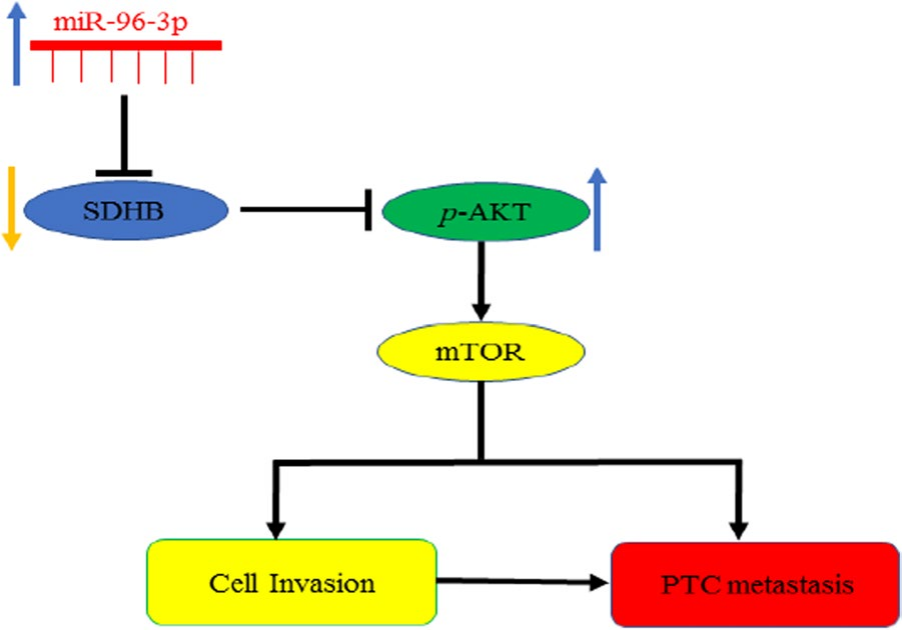
Schematic illustration of the relationship between miR-96-3p and papillary thyroid cancer. MiR-96-3p promotes the migration and invasion of papillary thyroid cancer through SDHB/AKT pathway

Taken together, our studies suggest that upregulation of miR-96-3p promotes tumor invasion and metastasis of thyroid cancer via regulating the SDHB/AKT/mTOR pathway (Fig. 6). These results suggest that miR-96-3p could serve as a biomarker and potential therapeutic target for PTC patients.

## Conclusion

Our studies uncovered that upregulation of miR-96-3p promotes the invasion and metastasis in PTC in vivo and vitro. MiR-96-3p regulates SDHB/AKT/mTOR pathway which indicates the miR-96-3p may be a biomarker for PTC.

## Supplementary information

**Additional file1: Figure S1.** The expression of miR-96-182-183 cluster in PTC tissues. **Figure S2.** The cell proliferation and MMP-9 expression of TPC and K-1 cell lines. **Figure S3.** The protein level of mTOR in TPC-1 and K-1 cell lines. **Figure S4.** The tumor volume of different groups within 6 weeks. **Figure S5.** The relation of SDHB and miR-96-3p in PTC tissues.

## Data Availability

The datasets used and/or analyzed during the current study are available from the corresponding author upon reasonable request.
