# Supplementary material for: MicroRNA-96-3p promotes metastasis of papillary thyroid cancer through targeting SDHB
Source: Cancer Cell Int. 2019 Nov 12;19:287. doi: 10.1186/s12935-019-1003-y (PMC6852711; doi:10.1186/s12935-019-1003-y)
Supplement: Supplementary file 1 — Additional file1: Figure S1. The expression of miR-96-182-183 cluster in PTC tissues. Figure S2. The cell proliferation and MMP-9 expression of TPC and K-1 cell lines. Figure S3. The protein level of mTOR in TPC-1 and K-1 cell lines. Figure S4. The tumor volume of different groups within 6 weeks. Figure S5. The relation of SDHB and miR-96-3p in PTC tissues. [file 12935_2019_1003_MOESM1_ESM.docx]

**Additional information**

## MicroRNA-96-3p Promotes Metastasis of Papillary Thyroid Cancer through Targeting SDHB

**Xupeng Zhao MDS^1^, Yingjie Li PhD^2^, Yong Zhou PhD^1,#^**

**Additional method**

**Xenograft tumor model**

Twenty healthy nude mice obtained from the laboratory of Fourth Affiliated Hospital of China Medical University and randomly separated them into two group (n=10 per group). Next, the K-1 cells were subcutaneously inoculated into the animals and after two weeks, the miR-96-3p mimics and negative control were injected into implanted tumor and then tumor volume was calculated. All the animals were sacrificed after 6 weeks.

**Cell proliferation assay**

MTT assay was performed to detect cell proliferation. Briefly, cells (1x10^3^ cells/well) were seeded into a 96-well culture plate. After growing with miR-96-3p mimics or inhibitor for 72h, MTT was added into the plate and cells were incubated for another 4 h in the incubator and then the optical density (OD) was measured.

**Additional results**


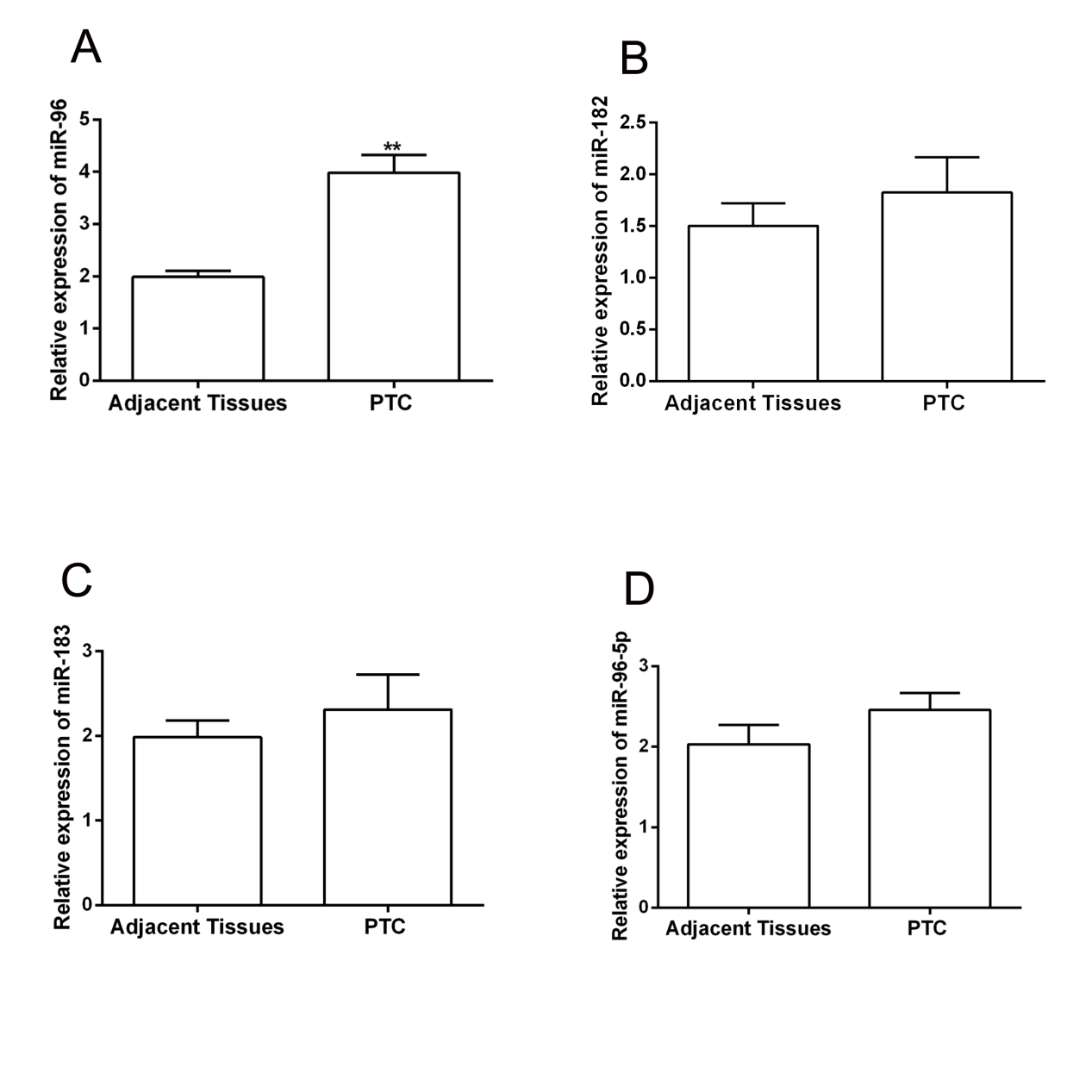


**Figure.S1. The expression of miR-96-182-183 cluster in PTC tissues.**

The relative expression of miR-96, miR182, miR183 and miR-96-5p in PTC tissues compared with adjacent normal thyroid tissues by real-time quantitative reverse transcription polymerase chain reaction (qRT-PCR) assay (n = 28; A-D). ***p* < 0.01 compared with adjacent tissues.


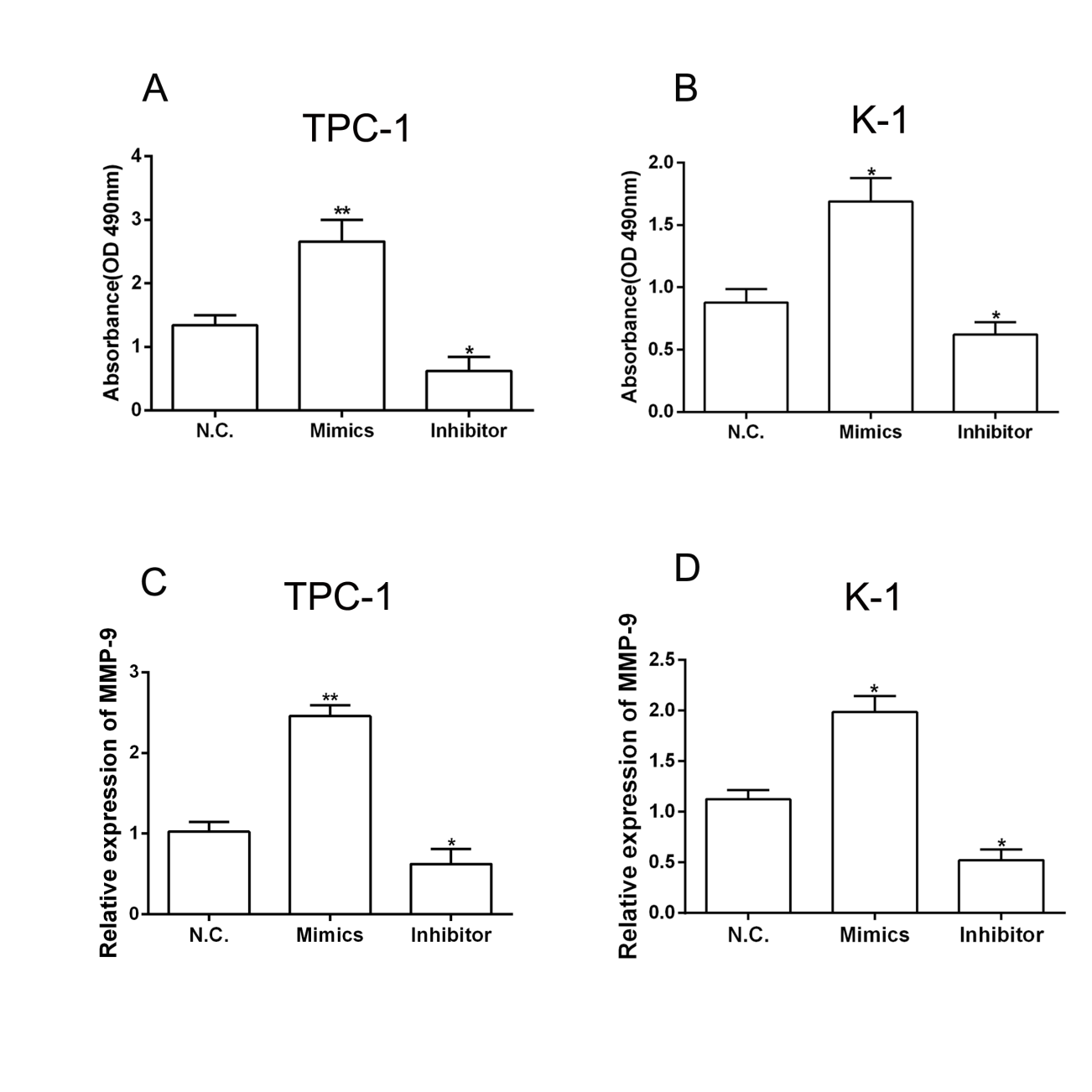


**Figure.S2 The cell proliferation and MMP-9 expression of TPC and K-1 cell lines.**

Cell growth of TPC-1 and K-1 cell lines after administration of miR-96-3p mimics and inhibitor (A and B). Relative expression of TPC-1 and K-1 cell lines after administration of miR-96-3p and inhibitor (C and D).**p*＜0.05，** *p*＜0.01 compared with negative control.


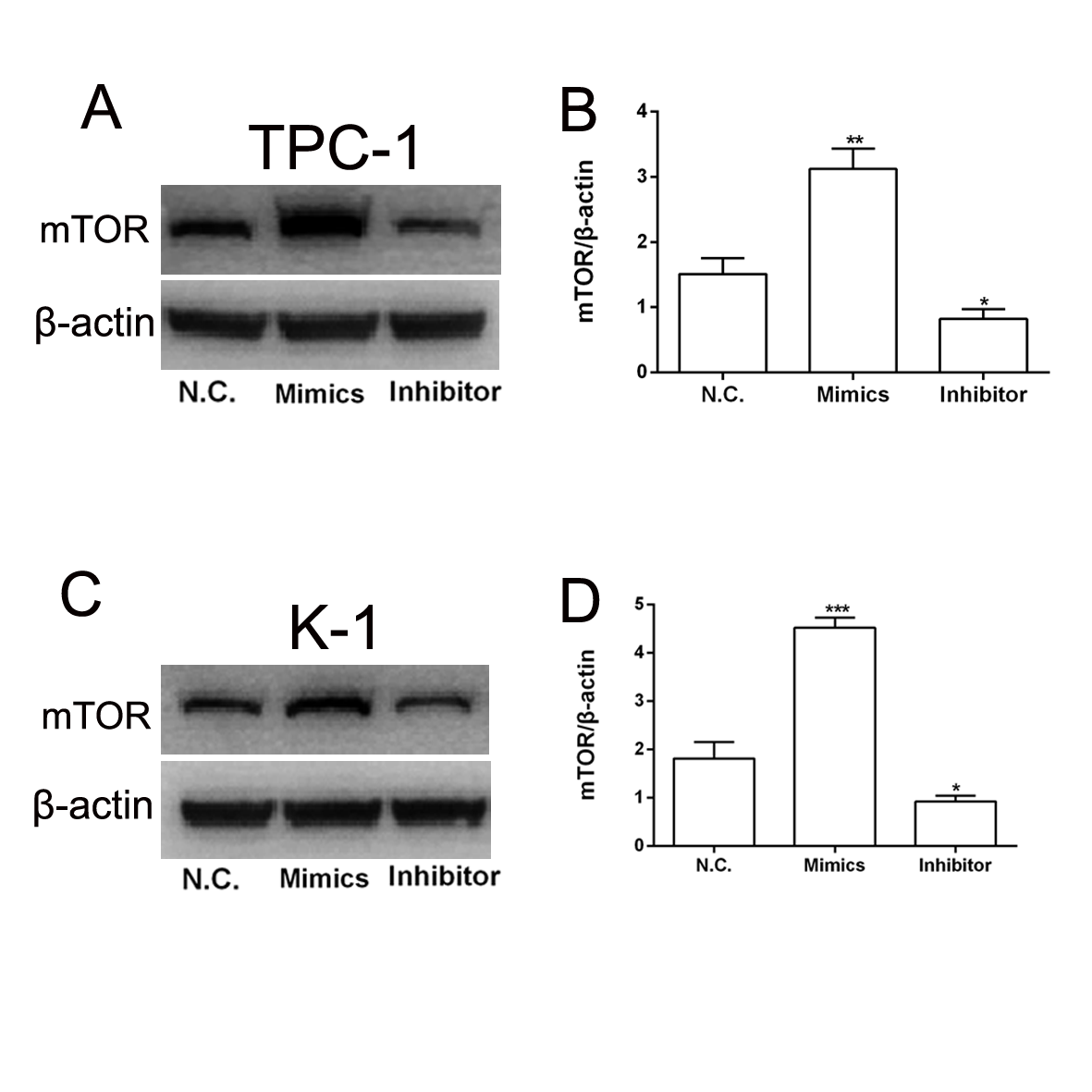


**Figure.S3 The protein level of mTOR in TPC-1 and K-1 cell lines.**

The expression of mTOR in TPC-1 cell line of different group after adminnistration of miR-96-3p minics and inhibitor. (A-B). The expression of mTOR in K-1 cell line of different group **(**C-D). **p*＜0.05，****p*＜0.001 compared with N.C. group.


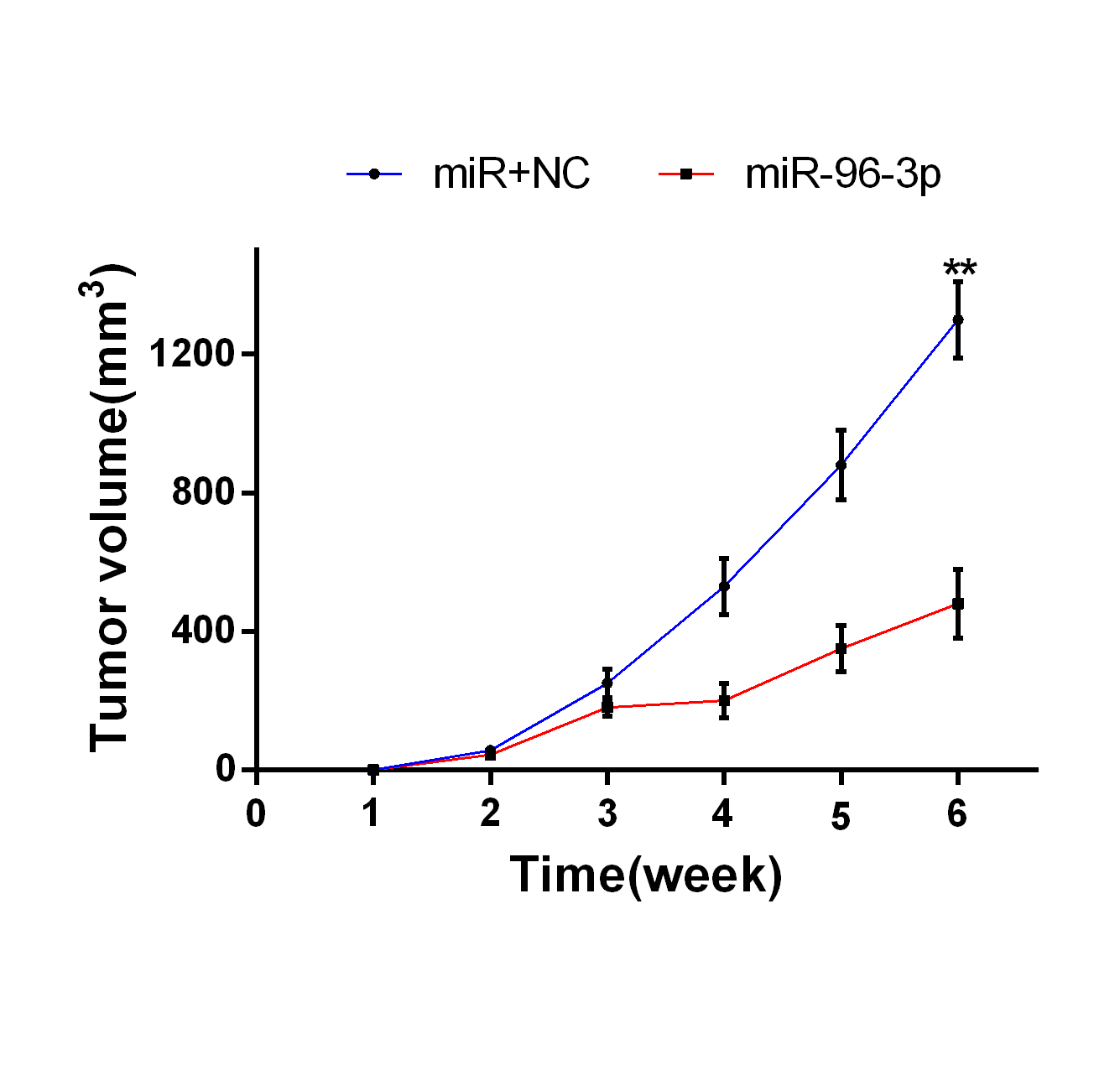


**Figure.S4 The tumor volume of different groups within 6 weeks.**

Tumor volume after performed with miR-96-3p and negative control. ***p*＜0.01 compared with miR+NC group.


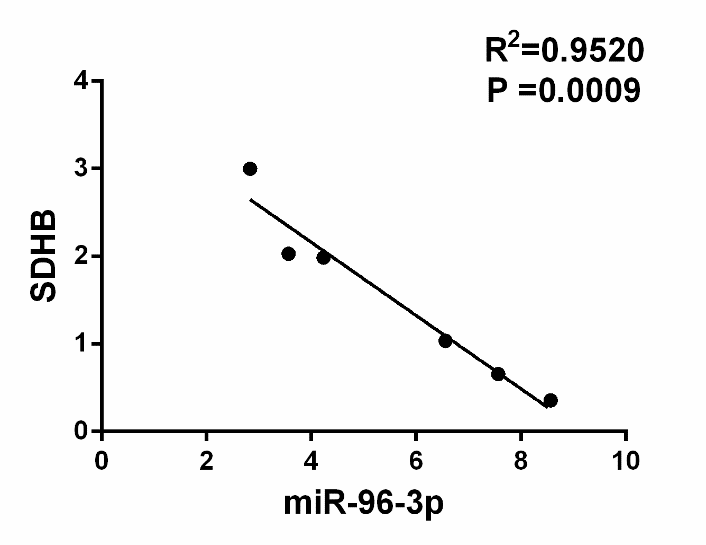


**Figure.S5 The relation of SDHB and miR-96-3p in PTC tissues.**

The relationship of SDHB and miR-96-3p expression relationship in PTC tissues.
